# Snake Cytotoxins Bind to Membranes via Interactions with Phosphatidylserine Head Groups of Lipids

**DOI:** 10.1371/journal.pone.0019064

**Published:** 2011-04-29

**Authors:** Anastasia G. Konshina, Ivan A. Boldyrev, Yuri N. Utkin, Anton V. Omel'kov, Roman G. Efremov

**Affiliations:** M.M. Shemyakin and Yu.A. Ovchinnikov Institute of Bioorganic Chemistry, Russian Academy of Sciences, Moscow, Russia; Université Joseph Fourier, France

## Abstract

The major representatives of Elapidae snake venom, cytotoxins (CTs), share similar three-fingered fold and exert diverse range of biological activities against various cell types. CT-induced cell death starts from the membrane recognition process, whose molecular details remain unclear. It is known, however, that the presence of anionic lipids in cell membranes is one of the important factors determining CT-membrane binding. In this work, we therefore investigated specific interactions between one of the most abundant of such lipids, phosphatidylserine (PS), and CT 4 of *Naja kaouthia* using a combined, experimental and modeling, approach. It was shown that incorporation of PS into zwitterionic liposomes greatly increased the membrane-damaging activity of CT 4 measured by the release of the liposome-entrapped calcein fluorescent dye. The CT-induced leakage rate depends on the PS concentration with a maximum at approximately 20% PS. Interestingly, the effects observed for PS were much more pronounced than those measured for another anionic lipid, sulfatide. To delineate the potential PS binding sites on CT 4 and estimate their relative affinities, a series of computer simulations was performed for the systems containing the head group of PS and different spatial models of CT 4 in aqueous solution and in an implicit membrane. This was done using an original hybrid computational protocol implementing docking, Monte Carlo and molecular dynamics simulations. As a result, at least three putative PS-binding sites with different affinities to PS molecule were delineated. Being located in different parts of the CT molecule, these anion-binding sites can potentially facilitate and modulate the multi-step process of the toxin insertion into lipid bilayers. This feature together with the diverse binding affinities of the sites to a wide variety of anionic targets on the membrane surface appears to be functionally meaningful and may adjust CT action against different types of cells.

## Introduction

One of characteristic features of cytotoxins (CTs) from snake venom is their ability to lyse different types of cells like erythrocytes, epithelial and certain lines of tumor cells [**1]**–[3****]. CTs are highly basic β-sheet proteins with molecular weight of about 6.5 kDa. All CTs have similar three-dimensional (3D) structures adopting a three-fingered loop-folding topology stabilized by four disulfide bonds. These proteins manifest strong amphiphilic properties on their molecular surface: apolar tips of loops I-III form a hydrophobic zone flanked by a positively charged “belt” composed of the conservative Lys and Arg residues. Experiments on model membranes [**4]**, [5] have demonstrated that the hydrophobic “bottom” represents a principal membrane-binding motif of CTs. Structural defects in lipid bilayer induced by CT binding to membrane have been demonstrated to lead to formation of the pore, whose size and life time have also been estimated [**6]**, [7****]. Despite the well-documented membrane-lytic activity and intensive studies of CTs (see [**2**] for review), exact molecular mechanisms of CT-induced cell damage are still unknown.

For a long time, it has been commonly accepted that CTs interact with cell membranes non-specifically and, therefore, have no particular targets in a lipid bilayer. The large body of experimental data accumulated so far, reveals a complex variety of CTs activities, including penetration inside cells and ability to interact with intracellular targets such as mitochondria [**8**] and lysosomes [**9**]. Moreover, CTs affect the action of important membrane binding proteins such as protein kinase C, Na^+^/K^+^-ATPase, and integrins [**2]**, [10]–[12****]. At the same time, possible targets mediating such a spectrum of CT activity still represent an intriguing challenge. NMR and X-ray data have demonstrated that CTs can bind low-molecular-weight ligands like heparin-derived oligosaccharides [**13]**–[15****], nucleotide triphosphates [**15]**, [16****] and sulfatide (SGC or 3′-sulfated β1-D-galactosylceramide) [**17**]. However, distribution of glycosphingolipids in membranes of various mammalian cell types differs extremely [**18**]. Taking into account the extensive profile of CT activity, along with the successful leakage experiments on anionic model membranes lacking sulfatides [**6]**, [19], [20****], it is reasonable to propose that CTs may specifically interact with other anionic membrane compounds as well. Here, the term “specifically” indicates binding of one or several lipid molecules (from the bilayer) to the particular sites on the CTs' molecular surface.

One of the putative targets for CT action is phosphatidylserine (PS). Being the most abundant anionic phospholipid in mammalian cells, it makes the largest contribution to the interfacial contacts with membrane-binding proteins and may affect their functioning [**21]**, [22****]. Earlier, it was shown that CTs can inhibit protein kinase C [**10**] and the authors suggested that CT binds to a site on PS that is also shared by protein kinase C. PS exposed on the cell surface is a hallmark of apoptosis and a signal for the removal of the cell by phagocytes [**23]**, [24****]. Despite the fact that PS is normally located in the inner leaflet of the lipid bilayer, various cellular events, including cell activation (activated blood platelets promote blood coagulation cascade), membrane fusion (myotube formation), cell division and aging also lead to PS exposure on the cell surface [**23]**, [25], [26****]. The externalized PS may accompany a variety of pathological states such as diseases, malignant transformation, cell injury and infection [**25]**, [27****]. Therefore, it seems reasonable to hypothesize that the presence of some portion of cells with “abnormal” PS exposure may provide the effective membrane “landing” of CTs.

In this work, we proposed a combined – experimental and modeling – approach to investigate possible role of PS in recognition and binding of CTs. Firstly, the membrane-permeabilizing activity of CT 4 from *Naja kaouthia* against phosphatydilcholine liposomes containing PS as a minor anionic component was assessed in fluorescent dye leakage experiments. Secondly, the search for the possible specific PS-binding sites on the molecular surface of CT 4 was performed using two independent molecular modeling techniques, namely, molecular docking and molecular dynamics (MD). As a result, three putative sites with different affinities to PS were found in the toxin molecule.

## Materials and Methods

### Leakage measurements

Synthetic 1-palmitoyl-2-oleoylphosphatidylcholine (POPC) was purchased from Avanti Polar Lipids (USA). PS and SGC were extracted from bovine brain in the Laboratory of Lipid Chemistry, Institute of Bioorganic Chemistry, Russian Academy of Sciences. CT 4 was isolated from *N. kaouthia* venom using the method described in [**28**] and finally purified by reverse phase chromatography. A stock solution of each lipid was prepared in ethanol/chloroform (1∶1) mixture. Liposomes of the following compositions were prepared: POPC, POPC/PS5%, POPC/PS20%, POPC/PS35%, POPC/PS50%, POPC/PS70%, POPC/SGC5%, POPC/SGC50% by mixing appropriate volumes of the stock solutions. After being dried under vacuum, lipid films were hydrated with 50 mM Tris buffer (pH 7.8) containing 30 mM NaCl, 4 mM EDTA and 100 mM calcein. After overnight incubation, the vesicle suspension was subjected to ten cycles of freezing/thawing and further extruded 20 times through polycarbonate filters with a pore size of 100 nm (Nucleopore, USA) using an Avanti mini-extruder (Avanti Polar Lipids, USA). Untrapped material was separated from the vesicles by gel filtration on a Sepharose CL-4B column with the elution buffer containing 50 mM Tris (pH 7.8), 110 mM NaCl, and 4 mM EDTA. For leakage measurements, a small volume of protein solution in Tris buffer was injected into a quartz cuvette containing the liposome suspension under constant stirring to obtain the lipid/protein ratio (L/P) = 100/1. In addition, the activity of CT 4 with respect to SGC-containing liposomes (POPC/SGC50%) was measured under the conditions described in [**7**] (buffer composition: 10 mM Tris-HCl (pH 7.4), 75 mM NaCl; 50 mM 6-carboxyfluorescein (6-CF) as fluorophore; L/P ∼62).

Release of calcein from liposomes caused by the addition of CT 4 was fluorimetrically monitored as a function of time on a HITACHI F4000 (Japan) spectrofluorimeter. Excitation and emission wavelengths were 494 and 517 nm, respectively. Percent of leakage (%L) was calculated according to the equation (%L) = 100(%)(*Ft*-*F*_0_)/(*F*_100_-*F*_0_), where *F_0_*, *Ft* and *F_100_* are fluorescence intensities recorded before addition of the protein, at time *t*, and after addition of Triton X-100 (0.5% final concentration, 100% leakage of calcein from the liposomes), respectively.

### Molecular modeling

**Figure 1:**
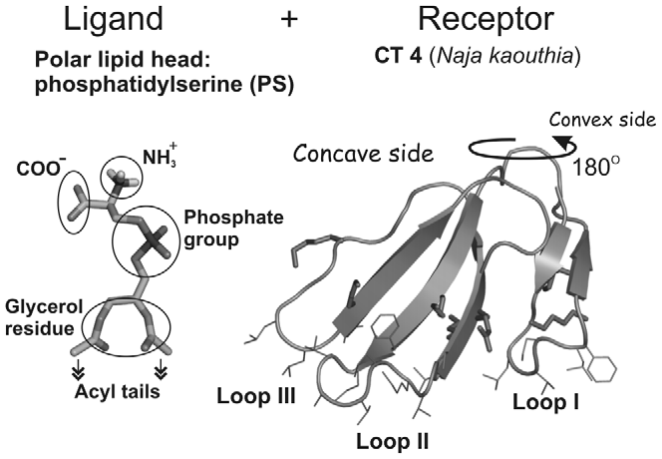
Three-dimensional structures used in molecular docking. Spatial model of PS head group (hPS) with two-carbon acyl moieties in place of the full-length acyl chains (“ligand”) is displayed on the left panel. X-ray structure of CT A3 from *Naja atra* (complete sequence analogue of CT 4 from *Naja kaouthia*) (“receptor”, right panel) is drawn in stick mode. β-Strands of CT are indicated with arrows. Side chains of the membrane-binding hydrophobic residues, along with the charged ones surrounding loops I-III, are shown. Functional groups of hPS are indicated.

All docking and MD calculations were performed with the short-chain PS (hPS) which contains two-carbon acyl tails in place of full-length acyl chains (**Figure 1**). Assuming the minimal involvement of lipid acyl chain region into the protein site recognition during the initial stage of CT binding, this choice of the ligand mimicking PS seems to be justified.

#### Molecular docking

Computational search for the putative PS-binding sites on the surface of CTs was performed using the GOLD 2.0 program [**29**] with the scoring function “goldscore” [**30**]. All rotatable bonds were sampled for the hPS moiety (hereafter called “ligand”), while the protein (“receptor”) was considered a rigid body. Unless otherwise specified, docking sphere with the radius of 30 Å was placed on the OH atom of the Tyr22 residue near the center of the convex side of CT 4. As a result, almost all the surface of the CT molecule was accessible to the ligand binding. Other parameters of the docking protocol corresponded to the default values implemented in the GOLD program. In total, 16 CT models (see below for details) were used in calculations. For each of them, at least 50 independent docking runs were performed and the results were ranked according to the “goldscore” value. Then, ten best docking solutions obtained for each model were combined into a so-called “TOP10” pool for further analysis.

Docking simulations were performed for the crystallographic structure of CT A3 from *Naja atra* (Protein Data Bank (PDB) entry 2BHI), full sequence analogue of CT 4 from *Naja kaouthia*. Also, the following calculated 3D models of CT 4 were used: (i) 11 spatial models of CT 4 obtained via 20-ns MD in explicit water; (ii) low-energy conformers resulting from the Monte Carlo (MC) search in the implicit hydrophobic slab mimicking membrane [**31**]. Other details of MC and MD computational protocols are described below and in Data S1.

hPS/toxin hydrogen bonds and ion contacts were evaluated with the PLATINUM [**32**] and GROMACS 3.3 [**33**] software. Molecular hydrophobicity potential distribution on the molecular surface of CT models was calculated as described elsewhere [**34**] using the PLATINUM program.

#### Molecular dynamics of CT 4 and CT 4-hPS in water

**Figure 2:**
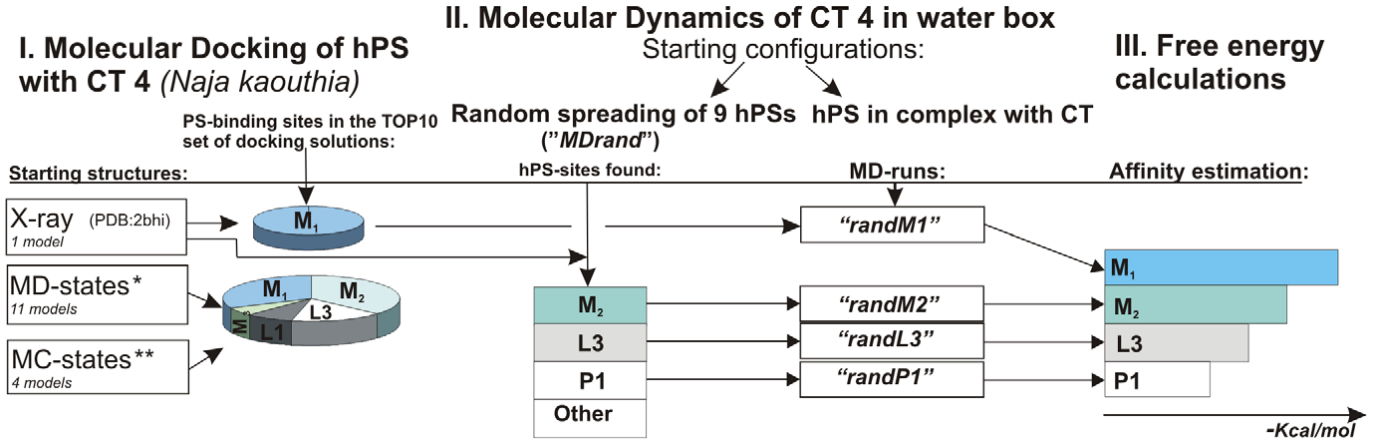
Prediction of hPS binding sites: block-scheme of molecular modeling approach and its main results. Three-dimensional (3D) models of CT 4 derived from molecular dynamics (MD) simulation in water and Monte Carlo (MC) search in a membrane-mimic media are marked with symbols “*” and “**”, respectively. The “TOP10” set of docking solutions contains 10 top-scoring docking poses found for every 3D model of the toxin (in total, 160 for all 16 3D models). hPS-binding sites identified in molecular docking and MD simulations (*MDrand*) are named (M_1_, M_2_, M_3_, L1, etc). Additional MD simulations of the complexes extracted from the TOP10 set (*randM1*) and *MDrand* trajectory (*randM2*, *randL3*, and *randP1*) were performed to estimate the binding affinities of hPS to the related toxin sites.

MD simulations were performed using the GROMOS96 force field [**35**] and the GROMACS 3.3 software [**33**]. The systems were set up and relaxed with the same computational protocol. The protein (in the case of *MDrand*, see **Figure 2**) or hPS-CT 4 complexes (all other MD runs) were placed in a rectangular box filled with ∼4000 SPC [**36**] water molecules. 3D periodic boundary conditions were imposed. To keep the system electrically neutral, counterions (Cl^−^) were added. Initially, the energy of the entire system was minimized using the steepest descent method. Then, the system was gradually heated from 5 K up to 300 K during 10 ps with approximately 10 intramolecular distance restraints between hydrogen-bonded backbone atoms of the β-structured core of CT 4. The obtained configuration of the system was taken as a starting one for MD production runs at 300 K in an NVT (constant volume and temperature) ensemble with a 2-fs time step. A double 10/12 Å cutoff and Particle Mesh Ewald (PME) algorithm (1.2 Å Fourier spacing) [**37**] were used to treat van der Waals and electrostatic interactions, respectively. The system was simulated during 15 ns out of which the last 10 ns were calculated without restrains. To minimize possible distortions of the toxin structure induced by hPS molecules randomly distributed in the water box (*MDrand* trajectory), distance restraints on the toxin molecule were preserved during all the simulation time (∼15 ns).

MD conformations for subsequent analysis were extracted with the interval of 5–20 ps from unrestrained parts of the MD trajectories. MD data processing was performed using a software developed by the authors and utilities supplied with the GROMACS package. The hPS-CT 4 complexes were visualized with PyMOL [**38**].

#### Monte Carlo conformational search for CT in an implicit membrane

To create 3D models of CT in the membrane-bound state, the X-ray and three MD conformations of CT 4 (Data S1) were subjected to MC simulations in the presence of an implicit “hydrophobic slab” membrane. The proteins' conformational space was explored *via* MC search in torsion angles space as described elsewhere [**39]**, [40****]. The implicit three-layer membrane model (water–cyclohexane–water) [**31**] based on the combined usage of atomic solvation parameters for nonpolar (gas/cyclohexane) and polar (gas/water) environments was employed. All-atom potential energy function for the protein additionally included the solvation energy term accounting for the influence of water membrane surrounding on the protein structure. All ionizable protein groups were taken in their charged forms. The starting conformations were arbitrary placed in the water phase. Non-bonded interactions were calculated using the spherical cutoff of 20 Å. Variable dihedral angles except for the ω ones were chosen randomly. Prior to MC simulations, structures were subjected to 80–150 cycles of conjugate gradients minimization. Then, about ten consecutive MC runs (5-10×10^3^ steps each) with different seed numbers and sampled 3, 2, 1 torsion angles (chosen randomly) were performed. At each MC step, the structures were minimized *via* 50–100 conjugate gradients iterations. In each run, the initial conformation was the lowest-energy one found in the preceding runs. In sum, up to ∼10^5^ MC steps were performed for each system in a single complete MC simulation. The calculations were carried out with the membrane thickness of 30 Å. Other details of simulations are given in [**31]**, [40****]. The resulting lowest-energy MC states obtained for each of the starting MD models were further used for docking of hPS.

#### Free energy calculations

To evaluate the binding free energies (Δ*G*) for hPS-CT 4 complexes, the potential of mean force (PMF) as a function of the toxin–ligand distance *r_AB_* was calculated. This was done by MD simulation in water using the constraint force calculation technique as it is implemented in the GROMACS program. Through a series of short MD runs (24 runs for each of the complexes), the PMF was calculated by integrating the mean force along the path AB, where initial state A is a bound state of hPS in a protein site while the final state B represents an unbound ligand in bulk water.

Initial structures of the hPS-CT 4 complexes (state A) were extracted from MD trajectories *randM1, randM2, randL3*, and *randP1* (**Table 1**). Each starting complex was solvated with SPC waters and subjected to energy minimization and heating for 10 ps. Chloride ions were added to make the systems electrically neutral. In the course of PMF calculations, the initial structure in each 50-ps MD window was generated *via* successive increase of *r_AB_* by 0.25 Å. Periodic boundary conditions and NVT ensemble at 300 K were employed. Electrostatic and van der Waals interactions were treated with the PME and cutoff methods, respectively.

**Table 1:** Binding free energies (*ΔG*) for hPS-CT 4 complexes.

Site of hPS binding	MD run*	Reference protein groups**	*ΔG* ***, *kcal/mol*
**M_2_**	***randM2***	Y11,F25,C42	−8.4±0.6
		K12,F25,C38	−7.2±0.2
		Y11,M24,I39	−6.8±1.0
**M_1_**	***randM1***	C3,M24,C42	−6.2±1.1
		N4,K23,M26	−6.1±0.5
		N4,K23,M26	−5.7±0.4
**L3**	***randL3***	F25,V41,C54	−5.0±0.4
		Y22,F25,C42	−4.3±0.4
		K23,C42,V49	−3.8±0.5
**P1**	***randP1***	C3,K35,K50	−3.8±0.6
		Y11,F25,C42	−3.9±0.5

*Starting hPS-CT 4 complexes were taken from the last 5 ns of 15-ns MD trajectories.

**defining the pathway from the complex to unbound hPS and CT 4 in calculations of PMF.

***Mean ± SEs are shown.

**Figure 3:**
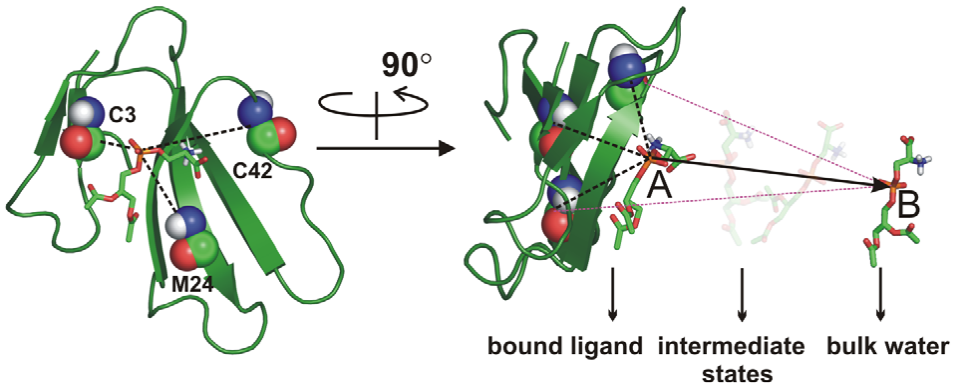
The scheme illustrating the choice of the reaction pathway (AB) in calculations of the free energy of CT-hPS binding. The potential of mean force (PMF) as a function of protein-ligand distance is calculated from the series of MD simulations of CT 4 - hPS complex in water. Initial (bound) and final (unbound) states of hPS are indicated by letters A and B, respectively. To provide the successful pulling direction (AB), backbone atoms of three residues (marked with a one-letter code) around the binding site were chosen. At every step (0.25 Å) of protein-ligand separation, the distances between the phosphorus atom of hPS and the center of mass of each of these protein groups were constrained and the mean force values were calculated and averaged over the ensemble of MD configurations derived from a series of short MD runs. hPS and CT 4 are drawn in stick and ribbon modes, respectively.

To define *r_AB_*, we used the P-atom of the hPS (the reference group) and backbone atoms of three residues of CT in the vicinity of a binding site (**Figure 3**). At every step, the distances between the center of mass of the reference group and each of the three protein groups were constrained and the constraint force was monitored. Since at each MD step the calculated mean force defines a direction for increasing *r_AB_*, selection of only one point in CT (center of mass for a group of atoms) often leads to “sliding” of the ligand (hPS) over the protein surface and not to its movement toward the bulk solvent (toward the state B). By contrast, selection of three points surrounding the binding site and located roughly equidistantly from each other allows efficient removal of the ligand into the solvent. Averaging the mean force values obtained at each step of the integration results in PMF curves, which describe the free energy changes upon the ligand binding. Using the one-dimensional radial PMF approach the equilibrium binding constants (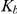) are calculated according to [**41**]



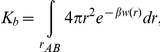
where *w(r)* is the total PMF of protein-ligand association, 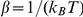. Then, ΔG values were defined from 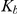 as 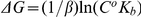, where 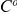 is the standard concentration (1 mol/L), 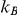 is the Boltzmann constant, and T is the absolute temperature. To assess reproducibility of the ΔG values for the same site, various orientations of the ligand in the site were chosen as starting conformations of the complexes. Also, several different sets of protein residues determining rAB were employed (Table 1).

## Results

### Fluorescence measurements: CT 4–induced leakage of PS-containing vesicles

**Figure 4:**
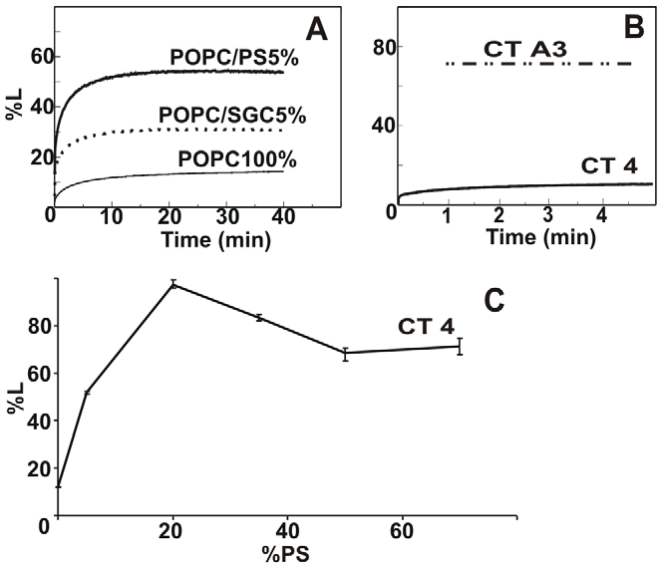
Effect of liposome composition on the efficiency of CT 4-induced leakage (%L) of fluorescent dye. **A** - CT 4 (Naja kaouthia)-induced release of calcein from POPC liposomes containing different anionic lipids: pure POPC, POPC/PS5%, and POPC/SGC5%. **B** - Kinetics of 6-CF fluorescence during the first minutes after CT 4 addition. For these measurements, the experimental conditions (buffer composition, fluorescent dye and lipid/protein concentrations in the measured volume) correspond to those in [**7**]. Stationary level of CT A3 (Naja atra)-induced leakage of 6-CF demonstrated in [**7**] is shown as (– .. –). **C** - Dependence of the CT-induced leakage efficiency on the PS content (in %) in POPC liposomes. Leakage values are calculated and averaged over 2–3 measurements.

To check the effect of anionic phospholipids (PS or SGC) on the ability of CT 4 to interact with bilayer membranes, we studied the toxin-induced leakage of fluorescent dye from the vesicles composed of neutral phospholipids (POPC) and small amount (5%) of PS or SGC. The results are given in **Figure 4A**. In accordance with the previously obtained data concerning the interactions of CT A3 with model lipid membranes [**6]**, [7****], CT 4 also caused no detectable lytic effect on pure POPC vesicles, whereas introduction of an acidic lipid into the model membrane led to a noticeable toxin-induced calcein release. As shown in **Figure 4A**, CT 4 displayed quite a pronounced effect on PS-containing vesicles increasing the leakage intensity up to 50%. Leakage was also produced in the POPC/SGC5% liposomes, although the observed effect was almost twice as low.

It is interesting to note that such a modest ability of CT 4 to induce leakage of the POPC/SGC5% liposomes contradicts the fluorescence data reported earlier for CT A3 [**7**]. Therefore, in order to minimize possible artifacts caused by differences in the experimental setup used in [**7**] and in this work, we also tested the leakage activity of CT 4 in the conditions similar to those described by Tjong et al. [**7**] (see Methods). Nevertheless, during the first minutes after the toxin addition, the extent of leakage was less than 10% against >70% in [**7**] (**Figure 4B**).

To get additional insight into the details of the leakage process, it was also investigated at different PS/POPC molar ratios. As a result, a bell-shaped dependence of the measured %L values on the concentration of the anionic lipid (5, 20, 35, 50 and 70%) was revealed. As shown in **Figure 4C**, the maximal efficiency of CT 4-induced leakage was observed in the POPC/PS20% liposomes. In this case, fluorescence intensity of the released calcein (L = 100%) is similar to that observed after treatment of the liposomes with detergent, thus indicating their complete destruction by the toxin.

### Search for PS binding sites on the CT 4 molecule using molecular modeling approach

Assuming that the hPS moiety may represent a target for CT 4 on the membrane/water interface, it was necessary to identify potential sites for the lipid head group binding on the molecular surface of the toxin. This was done using a number of computational experiments including molecular docking and molecular dynamics simulations.

#### Block-scheme of the computations

Block-scheme of the molecular modeling approach along with some principal results is depicted in **Figure 2**. First, the molecular docking procedure was implemented to recognize the protein binding sites for hPS. To take into account the protein flexibility, the standard docking scheme “flexible ligand/rigid receptor” was applied to a number of different protein conformations representing local conformational changes that characterize CT structures either in water or in membrane/water environment. Such conformations were generated by MD simulations of the toxin in explicit water and MC search with the implicit membrane model [**31**], respectively. Docking of hPS was performed to a number of MD and low-energy MC states of CT 4 as well as to its X-ray structure (see Methods).

Ten best docking solutions obtained for each 3D model of CT 4 (in total, 160 ligand-receptor complexes combined in the so-called “TOP10” set) and ranked according to the goldscore function were subjected to a stepwise clustering based on geometrical criteria. As a result, several hPS binding sites (named “M”, “L3” and “L1”) located in different parts of the CT molecule were identified. Depending on the exact position of hPS in the site (see below), the major group of solutions (site “M”) was subdivided into 3 clusters: M_1_, M_2_, and M_3_ (**Figure 2**).

One of the severe limitations of the docking protocol is that solvent effects are overlooked in the calculations. This may dramatically change the picture of protein-ligand interactions. That is why, to estimate reliability of the docking results, we performed several additional MD simulations of the toxin/hPS system in the explicit water. In this case, no *a priori* structural information about protein-ligand complexes was employed – in the beginning of MD simulations, nine hPS molecules were distributed randomly in the water box containing the X-ray model of CT A3 (PDB: 2BHI). In the course of the MD run (named “*MDrand*”), hPS molecules recognized the protein sites M_2_ and L3 that were already identified by the docking simulations mentioned above. In total, several hPS binding sites were delineated. One of them (site “P1”) has not been found previously with the docking procedure. Interestingly, it partially overlaps with the so-called “water-binding” omega-shaped region of loop II in P-type CTs [**42**]. Depending on the presence of the S28 (S-type of CTs) or P30 (P-type) conservative residue in loop II, CTs exhibit different functional activities [**43**]. Therefore, this potential PS-binding site was also kept in mind for further consideration. To assess stability of these systems and to avoid interference with other hPS molecules (e.g., competing for binding to neighboring regions of the molecular surface of CT), additional MD simulations of several complexes extracted from the *MDrand* trajectory were performed. For the protein sites M_2_, L3 and P1, the corresponding MD trajectories were named “*randM2*”, “*randL3*” and “*randP1*”, respectively. One more MD run in explicit water solution was carried out for the top-ranked hPS-CT 4 complex obtained by docking of hPS into the X-ray structure of the toxin and related to the M_1_ cluster. The corresponding MD-trajectory was called “*randM1*” (**Figure 2**).

The aforementioned MD-data (“*randX*”, where *X* = *M_1_*, *M_2_*, *L3* and *P1*) were used to estimate the affinity of hPS to the related sites on the protein surface. The corresponding free energy values were calculated for the hPS-CT complexes that remained stable during the MD simulation time (at least 5 ns).

#### Molecular docking of PS head group to CT 4

Combination of the docking runs carried out for 16 targets (**Figure 2**) resulted in three major sites for hPS binding on the molecular surface of CT 4. Solutions were found concentrated on the convex (site M) and concave (sites L1 and L3) CT surface sides, respectively. The most representative cluster of solutions, site M, was observed in more than 75% of the solutions from the TOP10 list (see Methods). Two alternative sites were identified only in few of the 16 analyzed 3D models of the toxin. Among the TOP10 solutions, clusters L3 and L1 represent 17% and 10%, respectively. It should be mentioned that sites L1 and L3 were not found among more than 100 docking runs performed for the X-ray structure of CT 4. The putative PS-binding sites and the related families of docking solutions were analyzed in terms of the “strength” of intermolecular hydrogen-bonding and ion contacts.

**Figure 5:**
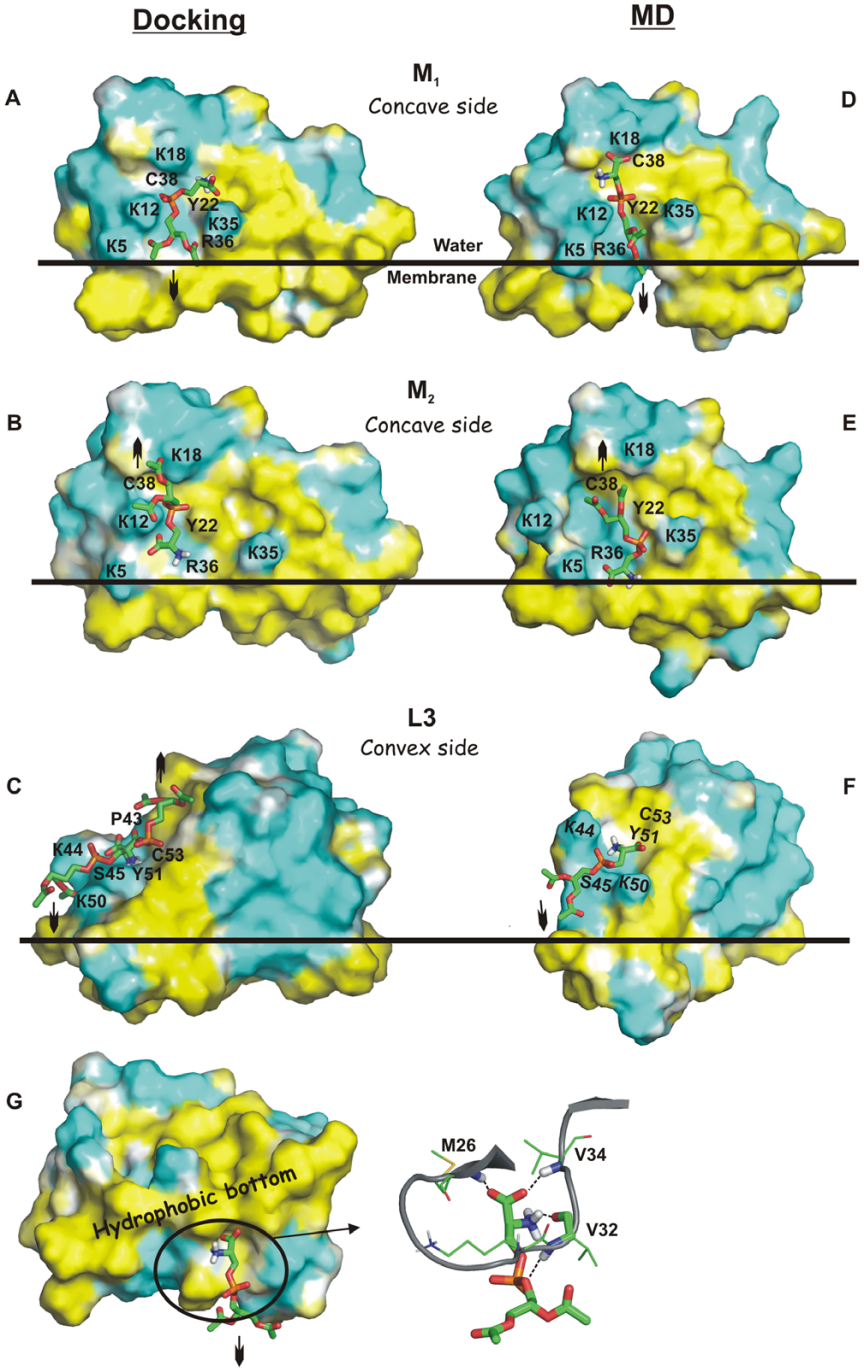
Proposed hPS binding sites on CT 4: results of docking (A–C) and molecular dynamics (MD) simulations (D–G). Molecular representation of the hPS-targeting sites M_1_ (**A, D**), M_2_ (**B, E**), L3 (**C, F**), and P1 (**G**). hPS molecules are shown in a stick representation. The site-forming residues are marked with a one-letter code. Hydrophobic and hydrophilic solvent-accessible surfaces of CT molecules are colored yellow and cyan according to the molecular hydrophobicity potential (MHP). Position of CT 4 relative to the water-membrane interface (horizontal line) corresponds to the proposed mode of CT binding to lipid bilayer [**5**]. Orientations of hypothetical full-length acyl chains of the ligand are marked by arrows. The water-capturing loop II of CT molecule that can bind hPS in the site P1 (**G**, view from the membrane-water interface) is shown in a ribbon representation (**G**, on the right). H-bonding in site P1 is displayed as a dashed line. The surface MHP was calculated and displayed using the PLATINUM [**32**] and PyMOL [**38**] tools.

Almost all residues in the M site are conservative. The lysine-rich cluster (Lys5, Lys12, Lys18 and Lys35) surrounds the polar surface that is formed by the backbone atoms of Leu6, Arg36, Gly37, Cys38 and OH-group of Tyr22 (**Figure 5AB**). This cluster usually contacts NH_3_^+^, COO^−^, and phosphate groups of hPS (**Figures 5AB** **,** **6AB**). As the molecular surface of hPS is mainly polar, the major factors driving formation of its complexes with the toxin are hydrogen bonds and electrostatic interactions. A characteristic feature of this binding site is the multivariant spatial arrangement of the ligand: average all-atom root-mean-square deviation (RMSD) value for the bound hPS is ∼7 Å. Detailed analysis of the TOP10 set of docking solutions revealed about 25 different types of intermolecular hydrogen bonds formed in the resulting hPS-CT 4 complexes. Furthermore, long flexible side chains of the charged lysines in the M site allow them to accommodate hPS in different orientations. Consequently, only two spatial models of CT 4 (with the extreme positions of Lys12 against Tyr22-OH) among the inspected 16 ones were not found in the relevant TOP10 solution list.

Detailed analysis of the docking poses within the site M revealed two main clusters nearly equal in size designated as M_1_ and M_2_. In these clusters, hPS moieties are oriented in the opposite directions with respect to the protein. Moreover, assuming that CTs bind to membranes *via* the apolar extremities of their loops I-III [**4]**, [5], [7], [44****], the toxin molecules have well-defined disposition with respect to the lipid bilayer. Therefore, orientation of hPS moieties in the protein site can be unambiguously associated with the position of hPS relative to the membrane. Taking this into account, we conclude that in cluster M_1_ hPS molecules are oriented in such a way that their acyl chains are immersed into the bilayer hydrophobic core (**Figure 5A**). On the contrary, the supposed long acyl chains of hPS of cluster M_2_ must be turned out from the bilayer interior (**Figure 5B**).

**Figure 6:**
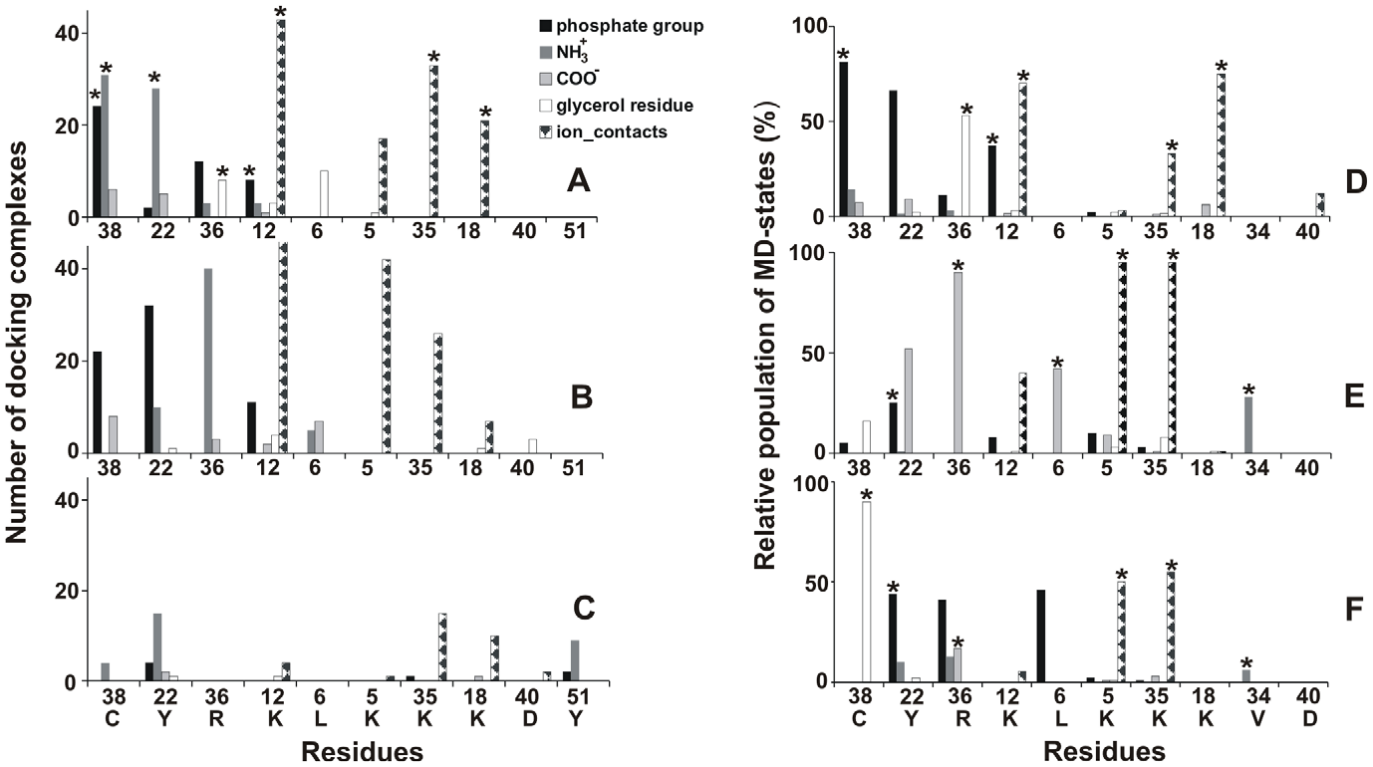
Distribution of H-bonds and ionic contacts between CT 4 and hPS in the M site.

Despite different orientations of the hypothetical hydrocarbon chains of PS, most of the docking solutions from the clusters M_1_ and M_2_ demonstrate similar patterns of hydrogen bonding with the toxin (**Figure 6AB**). Most of them are formed between 3–4 key residues (Lys12, Tyr22, Arg36 and Cys38) and the phosphate and/or NH_3_^+^-groups of hPS. Usually, these hPS-CT complexes are stabilized by 3 or more protein-ligand H-bonds. For example, the top-ranked hPS docking pose obtained for the X-ray model of CT A3 revealed five intermolecular H-bonds (**Figures 5A** **,** **6A**).

In addition to the two aforementioned clusters M_1_ and M_2_, a minor group (named M_3_) of docking solutions (about 10% of all poses in the site M) was isolated basing on the pairwise RMSD values calculated for the phosphate group of hPS. In this set of solutions, the hPS molecule is shifted from the central part of site M towards the Tyr51 residue and, in most cases, H-bonds between Tyr51-OH and phosphate or NH_3_^+^-groups of hPS are formed (**Figure 6C**). Also, glycerol moiety of hPS is preferentially directed toward the apolar tips of loop III although other orientations are possible as well. In contrast to sites M_1_ and M_2_, hPS in the M_3_ cluster demonstrates weaker ionic contacts with residues of the so-called lysine cluster, particularly Lys5 and Lys12 (**Figure 6C**).

Similar to the M site, accessible surface of the L3 site is mostly polar since it is formed by the backbone atoms of the conservative residues Pro43, Lys44, Lys50, Tyr51, Cys53 and Ser45 (including the side chain of the last residue) (**Figures 5C** **,** **7A**). In the TOP10 set of docking solutions, 26 of 160 complexes contain hPS bound in the L3 site.

**Figure 7:**
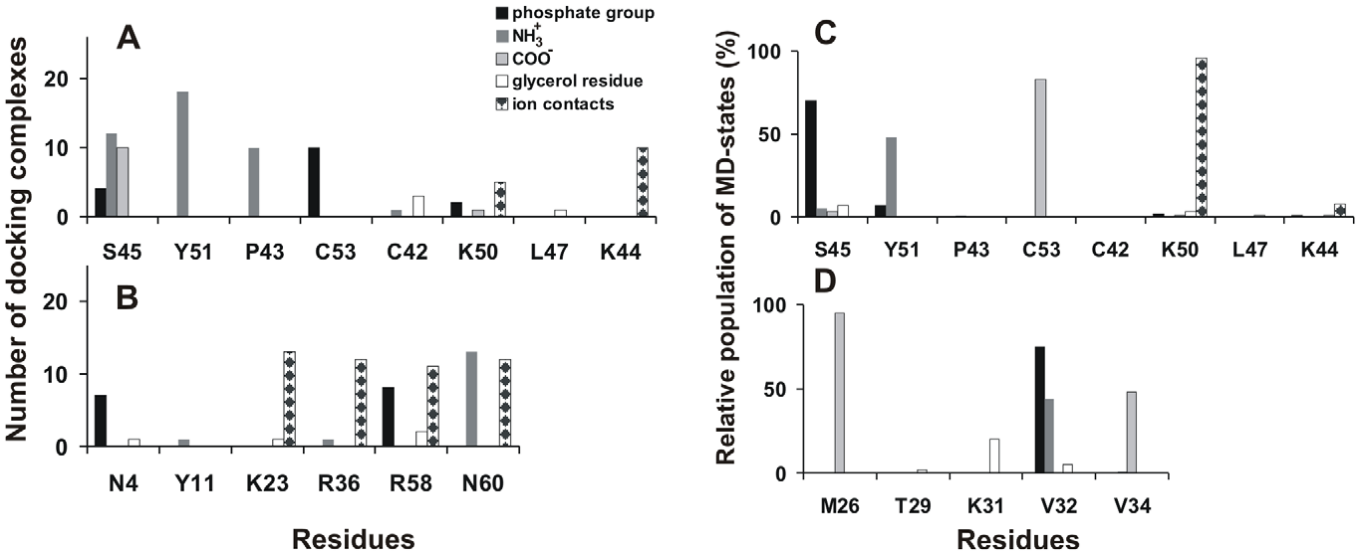
Distribution of H-bonds and ionic contacts in hPS-CT 4 complexes in sites L3, L1 and P1. Docking solutions from clusters L3 (**A**) and L1 (**B**), as well as MD complexes from MD simulations *randL3* (**C**) and *randP1* (**D**), are presented. Both *randX* MD trajectories were analyzed on the interval 5–15 ns. Other details are similar to those described in the legend to Figure 6.

In most of these docking solutions, the toxin residues form at least three H-bonds with NH_3_^+^ group of hPS (**Figure 7A**). As a result, structural plasticity of the bound ligand is less pronounced in comparison with the M site: in the whole set of docking poses average RMSD value for hPS atoms is ∼5.5 Å. Phosphate group of the ligand can be located either near the Cys53 or between Ser45 residue and side chain of Lys50 (**Figure 5C**). Assuming the membrane-bound state of CT (see above), these two positions of the phosphate group designate the opposite orientations of the glycerol moiety of hPS relative to the hypothetical membrane surface (**Figure 5C**). In docking solutions where the short-length acyl chains of hPS are oriented toward the membrane, side chains of Lys44 and Lys50 resemble a peculiar “pincers” capturing the phosphate group in the site. Similarly to the M site, the majority of solutions for this site have 4 and more protein-ligand H-bonds.

Another putative site of hPS binding – site L1 – was observed only for a small (13 from 160) group of docking solutions from the TOP10 set. This site represents a highly positively charged region located on the concave surface of CT 4 near the tips of loops I and II. It is formed by residues Asn4, Lys23, Arg36, Arg58, and Asn60. Solvent exposure of the bulky charged side chains of these lysines and arginines facilitates binding of an anionic lipid head group *via* attractive electrostatic forces (**Figure 7B**), although these interactions are not explicitly taken into account in the applied docking algorithm (GOLD software [**29**]). In all docking solutions related to the site L1, NH_3_^+^-group of hPS forms an H-bond with the C-terminal carboxyl group of residue Asn60. Similarly to sites M and L3, hPS bound in the L1 site is quite flexible and demonstrates different orientations of its glycerol moiety. In the considered complexes, no hydrogen bonds of hPS with the protein backbone atoms are observed. Therefore, due to the overall flexibility of the CT structure and, especially, the side chains mobility as compared with its backbone atoms, the site L1 may substantially change its shape and size.

#### Molecular dynamics simulations of CT 4 and hPS in water

Analysis of the results obtained *via* MD simulation (*MDrand*) started from a system containing the X-ray structure of CT A3 and nine hPS molecules randomly placed in a water box (**Figure 2**), leads to the following conclusions. Two of the ligand molecules quickly (within first 2 ns of MD calculation) recognized sites M and L3 on the toxin surface. Moreover, these hPS molecules preserved their interactions with the corresponding CT residues during all the remaining simulation time. Although other hPS molecules also revealed contacts with the toxin's molecular surface, no preferential distribution near particular protein sites was observed. Most of hPS molecules have no strong H-bonding with protein residues. The only exception is provided by hPS bound in the M site – during at least one third of its residence time in the site it forms more than three H-bonds with CT. Obviously, the presence of several ligands in solvent near the toxin molecule may change to some extent the parameters of their interaction with the protein sites. In order to eliminate such unwanted effects for hPS targeting sites M and L3, two additional MD runs - *randM2* and *randL3* - were performed (**Figure 2**). We should also note that no complexes similar to the M_1_ cluster of docking solutions where PS is supposed to expose its acyl chains towards a hypothetical lipid bilayer were found in the *MDrand* trajectory. Most probably, this is caused by insufficient MD sampling. Anyhow, to check the dynamic behavior of such complexes, MD simulation (*randM1*) was performed starting from the best docking solution of group M_1_ obtained for the X-ray structure of CT A3 (**Figure 2**). Below, we consider the results obtained for sites M and L3 in the set of simulations: *MDrand/randM2, randM1 and MDrand/randL3*.

In all MD runs, hPS preserves its starting orientation that corresponds either to the cluster M_2_ (in *MDrand*/*randM2* (**Figure 5E**)) or cluster M_1_ (in *randM1* (**Figure 5D**)) from the TOP10 set. At the same time, position of the ligand within the site is not well-defined - average all-atom RMSD values for hPS are in the range of 3–4 Å for all the MD runs. Furthermore, for approximately 90% of MD states orientation of hPS in the site deviates by 3–8 Å (in terms of RMSD) from that found *via* docking calculations. Such high ligand mobility observed in the course of MD is accompanied by rearrangements of hPS-CT intermolecular H-bonding (**Figure 6D–F**). The most stable H-bonds are formed between the phosphate group of hPS and residues Lys12 and Tyr22 through their side chains and Leu6, Arg36 and Cys38, through the backbone NH. As seen in **Figure 6D–F**, other functional groups of hPS are effectively involved in H-bonding. For example, glycerol moieties of hPS reproduce well their interaction with the NH-groups of Arg36/Cys38 along the whole *randM1/randM2* trajectories. Finally, in the course of *MDrand/randM2* simulations an additional Val34-CO – NH_3_^+^-hPS H-bond which was absent in docking solutions is also formed. In at least ∼25% of MD-states, the hPS molecule is stabilized in the site by four and more H-bonds with residues of CT 4.

Also, the electrostatically favorable interactions of phosphate/COO^−^ groups of hPS with the lysine cluster (residues 5, 12, 18 and 35) are reproduced quite well in the course of MD simulations. Obviously, strong or weak involvement of certain residues from the cluster into such ionic contacts depends on the orientation (M_1_ or M_2_) of hPS in the site (**Figure 6D–F**).

In both MD trajectories (*MDrand/randL3*), the phosphate group of hPS is located between the charged side chains of lysines 44 and 50 and forms stable H-bond with the OH- or NH-groups of Ser45 (**Figures 5F** **,** **7C**). The same H-bonds are found in some docking complexes related to the cluster L3. These solutions and all MD states have similar orientation of their glycerol moieties toward the supposed membrane core. Strong coordination of NH_3_^+^/COO^−^ groups of hPS by the backbone atoms of residues Tyr51 and Cys53 is observed along the whole *randL3* MD run (**Figure 7C**). It is also partly reproduced in the cluster L3 (**Figure 7A**). Moreover, for few MD-states from the trajectory *randL3*, hPS poses in this site are quite similar to those found in two of the docking solutions (RMSD for ligand molecule is ≤2.5 Å). Meanwhile, similar to MD states of hPS in the site M, *MDrand/randL3* simulations also produced a set of different positions of hPS in the site (4 Å in terms of average all-atom RMSD values).

Among hPS molecules derived from *MDrand* and possessing intensive hydrogen bonding with the protein, one is bound near the tip of loop II (site P1). The site contains the backbone atoms of Met26, Val32, and Val34 and side chain of Lys31 (**Figure 5G**). It includes both the membrane-binding motif of CT and the water-binding site in the loop II [**42**]. Particularly, in the water-binding site the amino group of the conservative Met26 is an invariable donor of hydrogen in H-bonds with oxygen of water. In site P1, NH-group of Met26 also forms a stable H-bond with the carboxyl group of hPS (**Figure 7D**). Moreover, the backbone atoms of the water-binding residue Val32 form H-bonds with phosphate and NH_3_^+^ groups of hPS (**Figure 5G**). Analysis of MD trajectory *randP1* shows that hPS, excluding its glycerol moiety, occupies quite a fixed position in the site (at least during the first 7 ns). The average value of RMSD over the head group of hPS is only 1.5 Å from the starting conformation. During the next several nanoseconds of *randP1*, hPS leaves the P1 site.

#### Free energy calculations

**Figure 8:**
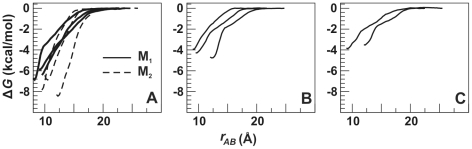
Free energy (Δ*G*) changes upon hPS-CT 4 association. Total potential of mean force (PMF) as a function of hPS-CT 4 distance (*r_AB_*) (see Methods for definition of *r_AB_*). PMF curves for hPS-binding sites M_1_ and M_2_ (**A**), L3 (**B**), and P1 (**C**) are shown.

To compare the strength of hPS binding to different CT 4 sites described above, we estimated corresponding free energies of binding (Δ*G*) based on PMF data (see Methods). The results obtained for the complexes predicted by docking (site M_1_) and by MD simulations (sites M_2_, L3, P1) are given in **Table 1**. To test reproducibility of the calculated values of Δ*G*, a number of simulations were done for different MD complexes as well as for the alternative sets of “reference protein points” defining the reaction path in calculations of PMF. The one-dimensional PMF profiles are presented in **Figure 8**. The minimal values of PMF as well as the calculated free energy (**Table 1**) depend moderately on the exact structure of the complex and the choice of the reference points, although variations in the resulting Δ*G* within each site are meaningfully less than those between the sites.

The strongest binding of hPS to the toxin was observed in the M_2_ site - on average, the corresponding values of Δ*G* were approximately 1.5, 3.1, and 3.6 kcal/mol lower than those obtained for sites M_1_, L3, and P1, respectively (**Table 1**). Therefore, according to the predicted affinities for hPS, the sites in CT 4 may be ranked as follows: M_2_ > M_1_ > L3 > P1. A putative reason for the weak binding of hPS in the P1 site is the remoteness of the positively charged protein side chains from the site. Noteworthy, orientation of hPS in site M_2_, where the hypothetical acyl chains of the ligand are turned away from the membrane, seems to be more energetically favorable than the opposite one (acyl chains directed toward the membrane).

## Discussion

Numerous attempts have been made to determine the molecular mechanism of CT's action. Previous chemical modification studies have revealed that none of CTs' loops is exclusively responsible for biological activities of the toxins [**45]**–[47****]. Instead, membrane penetration of CTs is likely to be determined by the continuous hydrophobic stretch formed by the tips of loops I-III [**4]**, [5], [7], [44****]. In view of complexity of the system, molecular details of membrane recognition by CTs are still poorly understood. Usually, they are limited to consideration of correlations between hydrophobic and electrostatic constituents of interaction between the CT and lipid bilayer.

Thin-layer chromatography and fluorescence spectroscopy measurements have previously demonstrated that CTs can bind negatively charged lipids and induce membrane leakage by making pores in charged vesicles, whose size and life time have also been estimated [**6]**, [7], [19], [20****]. Existence of a specific binding site on the toxin molecule is confirmed by X-ray data [**17**]. In the crystal structure of CT A3 dimer, a well-defined anion-binding pocket on the convex side of the toxin effectively binds the head group of sulfatide. This sphingolipid is known to be located in the outer leaflet of the plasma membrane [**18**]. Based on this observation, much attention has been paid to SGC as a potential membrane target for CT A3 and a number of successful experiments on rat cardiomyocytes have been carried out [**17**]. However, the complexity of such *in vivo* system usually makes the interpretation of the measured effects at molecular level too difficult. In this situation, experiments on simple model systems are required. Unexpectedly, our fluorescence-leakage experiments with the full sequence analogue of CT A3, toxin CT 4 from *Naja kaouthia*, did not demonstrate significant lytic effect on the lipid vesicle preparations similar to those used in [**7**]. (It should be noted that 6-CF fluorescent dye used in [**7**] was proved to be capable of spontaneous leakage from liposomes [**48**].) On the contrary, substantial calcein release from PS-containing vesicles was demonstrated.

As shown in this work, the lytic activity of CT 4 increases drastically for POPC/PS20% liposomes (∼100% calcein release). To explain the observed effects, we estimated the number of PS moieties per toxin molecule in liposomes of the given composition. Basing on simple geometrical criteria (surface areas occupied by a lipid head group and CT in the membrane-bound state, liposome diameter of 1000 Å, and L/P ratio of 100) and supposing uniform distribution of PS in lipid membrane and complete binding of CT 4 to vesicles at a given L/P ratio, we found that at least three PS molecules bind to each toxin molecule in POPC/PS20% liposomes. The drastic decrease in lysis efficiency for POPC/PS5% liposomes indicates that the mechanism determining the binding of the highly basic toxin to the anionic membrane includes nonspecific electrostatic attraction. Indeed, in the case of POPC/PS5% liposomes, charge compensation occurs at very low CT concentrations (L/P ∼300); therefore, at a higher toxin content (L/P = 100), some CT molecules are not capable of membrane binding. However, even in the case of complete CT binding to the surface of POPC/PS5% liposomes, charge density of the membrane is too low: on average, there are only 0.8 PS molecules per surface area in contact with the toxin molecule. As a result, CT does not embed efficiently into the bilayer. On the other hand, the observed decrease in CT 4 activity with respect to liposomes at PS content >35% is most likely caused by changes in the bilayer properties, in particular, by an increase in lipid packing density near the interface [**49**].

To identify specific binding sites for anionic lipid head groups of PS on the surface of CT 4, two molecular modeling approaches were used. As a result, three hPS binding sites - M, L3, and L1 - were found in the CT 4 molecule. The site M was supposed to be the most reliable one for hPS binding. Firstly, it is identical to the binding site of SGC observed in the X-ray structure of CT A3 [**17**]. Secondly, hPS molecule binds the M site in most of the independent docking and MD simulations. In such complexes, hPS reveals the greatest number of energetically favorable contacts with the toxin and demonstrates the lowest free energy of binding. Finally, basing on the mutagenesis data obtained for toxin γ (*Naja nigricollis*) [**45]**, [46****], it was suggested that the residues of the M site should be involved in the interactions with phospholipids [**50**].

In contrast to the M site, the existence and possible functional meaning of the newly recognized site L3 are not so obvious. Being located on the concave side of the toxin, far from the known membrane-binding motif, this site is less accessible to PS head groups. On the other hand, some data indirectly show that a certain region of loop III is likely to have a functional importance. Thus, based on the observed correlations between variations in amino acid sequences of CTs and their depolarization activities on cultural skeletal muscle, Hodges et al. [**51**] revealed the importance of some residues, including Lys44 and Lys50 attributed here to the L3 site. Furthermore, a consensus sequence L/PKSSLL, which mimics an epitope on the loop III of CT A3 (including Pro43, Lys44, and Ser45 of the L3 site) was recently identified as a crucial neutralizing epitope for antivenin binding [**52**]. However, we should mention that in many cases the functional importance of particular residues observed in experiments does not mean that these residues are necessarily involved in lipid binding. Obviously, delineation of the related intimate mechanism of the toxins' action requires future investigation.

The third predicted hPS-capturing region of CT 4 includes a strongly polar zone at the concave surface of the loops I-II (site L1) and the hydrophobic “bottom” of the loop II (site P1). Structural features of the site L1 differ from those of sites M and L3. Also, this region of the molecule is mentioned as a place for ATP binding [**16**]. The site P1 practically overlaps with the site adopting the long-living water molecule. To date, the tightly bound water molecules were observed within the loop II of most of P-type CTs, whose structures were solved [**42]**, [53], [54****]. Also, it was proposed that this water molecule determines the omega-shaped conformation of loop II [**54**]. As a result, tips of all three loops form a combined hydrophobic “bottom”, the membrane binding motif of these CTs [**4]**, [5], [55****]. Similar to the coordinated water, hPS interacting with the P1 site stabilizes the omega-shaped conformation of the loop II. Altogether, this permits an assumption that hPS can “pick up the baton” from the long-living water to hold structural integrity of the membrane-binding platform. This, in turn, provides an additional “anchor” for the more powerful toxin-bilayer interaction.

We can therefore draw the following picture of CT-membrane interactions. The successful hPS-CT association is primarily determined by the long-range electrostatic and medium-range hydrophobic forces promoting the interface-inserted mode of toxin binding. This initial stage of protein-membrane interaction is followed by the specific short-range “trapping” of anionic lipid head groups into the particular toxin's sites. Based on the geometry of the calculated membrane-bound states of CT 4, one can see that all three sites differ in their accessibility to PS head groups of the bilayer. For instance, the cluster of basic residues of the L1 site is located in close proximity to water/membrane interface and, therefore, this site is probably targeted in the first place. On the contrary, residues of the M site, the main candidate for specific ligand binding, are relatively far from the bilayer and apparently should not interact with it. However, being located on the convex side of the membrane-bound CT 4 and directed toward the membrane interface, site M can be spontaneously occupied by an anionic lipid head group due to thermal fluctuations.

At the same time, we cannot exclude an alternative scenario: while approaching the bilayer, CT molecule initially adopts a “flat” orientation with its convex side directed toward the membrane surface, which is followed by hPS-CT complex formation. Then, the anchored toxin molecule changes its orientation due to deeper insertion of the hydrophobic tips of the loops into the lipid bilayer. In this case, the first player is the M site - strong electrostatic interactions of its residues with PS groups initially promote the complex formation. Occurrence of such contacts is confirmed by NMR measurements obtained for homologous CT A3 in SGC-containing dodecylphosphocholine micelles [**7**]. Thus, it was shown that the toxin embeds into micelles with all three hydrophobic tips of the loops, and binds SGC in the M site, near residues Val7 and Cys38.

The aforementioned mechanisms only take into account sites M and L1, as well as the auxiliary role of the P1 site. These assumptions are based on quite a solid experimental ground. In contrast, putative involvement of site L3 remains rather speculative because it is not supported by experimental data. We can only propose that this site works late in the toxin insertion, when CT molecule deeply penetrates into the membrane interior. In addition, an optimal PS content seems to be very important. Apparently, it ensures involvement of all three toxin sites in CT–membrane interaction.

Apart from establishing energetically favorable electrostatic contacts with cationic CTs, the presence of anionic lipids has another important impact. Thus, we have recently demonstrated that pure and binary lipid bilayers containing PS (or other anionic lipids), exhibit a prominent mosaic nature of their water-accessible surfaces [**56]**, [57]. In particular, they reveal dynamic hydrophobicity clusters created by acyl chains “snorkeling” near the interface. It was shown that in such clusters, lipid tails can efficiently interact with external agents, like membrane active peptides [**56**]. In this case, the binding process is driven by self-accommodation of amphiphilic peptides on the heterogeneous hydrophobic/hydrophilic membrane surface. The insertion is additionally facilitated by the formation of highly specific and long-living contacts between some of the protein side chains (especially, of Arg, Lys, and Trp) and anionic lipid heads [**56**]. We therefore propose that the binding of CTs to PS-containing bilayers is quite similar to that observed for cationic amphiphilic peptides – it is determined by specific electrostatic contacts between the sites in CTs and PS as well as by nonspecific hydrophobic interactions.

The former factor not only enlarges the CT-membrane binding constant, but also can promote putative reorientation of PS acyl tails in the binding sites of CT followed by oligomerization of the toxins. Thus, in the sites M and L3, hPS glycerol residue has two opposite orientations with respect to the membrane surface (see Results for more details). Earlier, similar picture has been theoretically predicted [**17**] and experimentally observed for SGC fatty acid chains in complex with CT A3 [**7]**, [17****]. This corroborates the computational approach used in this work. Unfortunately, we still can not answer the question how switching of the lipid tails occurs. Probably, the high energy cost of this process may be decreased *via* local perturbations of the membrane in response to the initial toxin binding in the interface-inserted mode. Also, it can be promoted by the presence of hydrophobic clusters on the PS-containing membrane surface where the lipid packing is weaker than in the rest parts of the bilayer.

There are obvious pitfalls in the current approach. First of all, they are related to the choice of PS as a target for CTs. Indeed, PS is mainly found in the inner leaflet of cell membrane. This fact has major physiological importance, especially for removal of apoptotic cells [**24**]. Besides this, the exposure of PS is also detected in response to some physiological stimuli as well as in senescent cells and at certain stage of cell development [**23]**, [25], [26], [58****]. Hemolytic and muscle depolarization activities of the snake venom CTs are the primary and reproducible tests for biological activity of CTs. It is known that red cell composition of bloodstream is dynamic and some (at least transient) portion of senescent, effete or injured cells with “abnormal” PS exposure exists. The values of exposed PS in human erythrocytes were estimated to be in the range from zero to few percent [**59]**, [60****]. Furthermore, it should be noted that PS content may differ between organisms. For instance, rat erythrocytes have increased PS fraction in the outer monolayer compared to the human ones [**59**]. Cell surface exposure of PS has also been shown to mediate myotube formation and to form cardiac muscle from myocardioblasts [**23]**, [58****]. Thus, in plasma membrane of chicken embryo fibroblast and myoblasts 20 and 45% of PS, respectively, is externally disposed [**61**]. So, at least in some of usual preys of the cobra hunt (like rats, bird eggs, *etc*.), blood and muscle cells contain small or even considerable amounts of PS on their outer membrane surface. This seems to be sufficient to initiate the effective membrane “landing” of CTs. Most likely, the optimal (for CT-membrane binding) anionic lipid content is also achieved due to the presence of other head groups such as various glycolipids and phosphatidyl inositol). The latter lipid is asymmetrically distributed in the membrane of the human red blood cells - its content in the outer leaflet may reach 20% [**62**]. Moreover, *in vivo* experiments have revealed that diverse anionic lipids (PS, cardiolipin, phosphatidyl inositol) added to a cell suspension were able to inhibit the CT A3 action [**63**]. This indicates the ability of toxins to accommodate their sites for binding of different lipid head groups. Furthermore, PS exposure in a target cell can be up regulated at the later stages of CT action (with still unclear molecular details) including deep membrane penetration, oligomerization, and pore formation. One of possible mechanisms is related to CT-induced increase of cytosolic Ca^2+^ - like in rat cardiomyocytes [**64**]. This, in turn, induces Ca^2+^-dependent activation of scramblase resulting in rapid PS appearance on the cell surface (within minutes for erythrocytes) [**65**].

These newly exposed PS molecules represent additional targets for CTs. At the further stages of CT action, internalized CT molecules can also bind to the inner-leaflet PS and affect some key enzymes such as protein kinase C [**10**] and so on till the full cell damage. It was shown that ATP release from lysed erythrocytes provides sufficient extracellular concentration of ATP (normally being quite low) to induce activation of a non-selective ion channel P2X_7_ in murine T cells and, as a consequence, leads to a cascade of events, including externalization of PS [**66**]. Increased PS exposure on cells adjacent to the lysed ones may be one of the ways to propagate CT-induced cell death. The drawn picture of CTs' action qualitatively explains the observed effect of “one after another” accumulation of fluorescently labeled CTs on the membrane of a particular cell followed by its lysis [**67**]. To summarize, the highly dynamic and heterogeneous PS exposure is quite usual for populations of blood, muscle, and other cells even in normal conditions. This seems to justify selection of PS as a target for CTs' action.

Another limitation of the current work is related to selection of the ligand structure used in computer simulations. In fact, instead of the entire PS molecule only its head group is considered, and the effects of membrane environment are not taken into consideration. Probably, omitting one or both of these factors can alter the simulation results. On the other hand, it seems quite reasonable to assume that the hydrocarbon tails of PS incorporated into a lipid bilayer are inaccessible to external agents (e.g., CTs) at the initial stages of protein-membrane recognition. It is important that most of the predicted results correlate well with the experimental data. For instance, overall reliability of the docking approach has been earlier confirmed in [**68**], where hPS has been used to detect PS-binding sites on phospholipid-binding proteins, kinase C alpha and GM2-activator. Finally, without these approximations, all-atom MD simulation of CT interacting with hydrated PS-containing lipid bilayer becomes computationally too expensive. In addition, complexity of the system and the lack of direct structural information about its initial configuration can lead to a situation, when weak effects are shielded by statistical, parameterization, and other errors. Taking into account the approximations made, this would overestimate potentialities of the method. In this work, we used a simplified model to delineate just the principal trends in the behavior of systems under study – without the aim to get precise structural information.

### Conclusions

Based on fluorescent measurements of CT 4-induced leakage of dye from lipid vesicles and computer simulations, a hypothesis about binding sites of PS head group in CT has been suggested. In particular, we assume that the well-defined anionic binding pocket on the convex side of CT, site M, as well as the newly identified site L3 on the concave side of the toxin, both have strong ability to accommodate low-molecular-weight compounds such as head group of PS in a number of different conformations. This feature together with diverse binding affinities of the sites to different anionic targets on membrane surface appears to be functionally meaningful and may adjust CT action against different types of cells. The delineated protein-lipid interactions can be considered as an initial step of the membrane permeabilization events. Like other membrane active agents (e.g., antimicrobial peptides [**69**]), CTs employ a wide arsenal of specific and nonspecific tools in order to accomplish their function – lysis of cell membrane. Different scenarios of binding of anionic lipid heads in the specific sites on the toxin surface together with the control over the formation of the “hydrophobic bottom” promoting anchoring of CTs on the membrane surface are among them. Mosaic nature of the membrane surface (mainly due to the presence of anionic lipids) may also contribute to mutual adaptation of the two amphiphatic systems (toxin and membrane). In our opinion, such a diversity of the factors important for toxin-bilayer interactions assures efficient and robust binding of CTs to cell membranes.

## Supporting Information

Data S1 **Spatial models of CT 4 used in molecular docking.** To take into account flexibility of the receptor (CT 4), docking of hPS was performed into a set of toxin conformations obtained *via* molecular dynamics (MD) and Monte Carlo (MC) simulations starting with the X-ray structure of CT A3 (PDB entry 1HOJ). Some details of MD protocol as well as the starting structures for MC search are indicated. Structural rearrangements of the toxin molecule in water (MD simulation) and membrane/water (MC search) environments are described.
(DOC)

## References

[1] Dufton MJ, Hider RC (1988). Structure and pharmacology of elapid cytotoxins.. Pharmacol Ther.

[2] Kumar T, Jayaraman G, Lee C, Arunkumar A, Sivaraman T (1997). Snake venom cardiotoxins-structure, dynamics, function and folding.. J Biomol Struct & Dyn.

[3] Feofanov AV, Sharonov GV, Dubinnyi MA, Astapova MV, Kudelina IA (2004). Comparative study of structure and activity of cytotoxins from venom of the cobras Naja oxiana, Naja kaouthia, and Naja haje.. Biochemistry (Mosc.).

[4] Dauplais M, Neumann JM, Pinkasfeld S, Menez A, Roumestand C (1995). An NMR study of the interaction of cardiotoxin gamma from Naja nigricollis with perdeuterated dodecylphosphocholine micelles.. Eur J Biochem.

[5] Dubovskii PV, Dementieva DV, Bocharov EV, Utkin YN, Arseniev AS (2001). Membrane binding motif of the P-type cardiotoxin.. J Mol Biol.

[6] Forouhar F, Huang WN, Liu JH, Chien KY, Wu WG (2003). Structural basis of membrane-induced cardiotoxin A3 oligomerization.. J Biol Chem.

[7] Tjong SC, Wu PL, Wang CM, Huang WN, Ho NL (2007). Role of Glycosphingolipid Conformational Change in Membrane Pore Forming Activity of Cobra Cardiotoxin.. Biochemistry.

[8] Wang CH, Wu W (2005). Amphiphilic beta-sheet cobra cardiotoxin targets mitochondria and disrupts its network.. FEBS Lett.

[9] Feofanov AV, Sharonov GV, Astapova MV, Rodionov DI, Utkin YN (2005). Cancer cell injury by cytotoxins from cobra venom is mediated through lysosomal damage.. Biochem J.

[10] Chiou SH, Raynor RL, Zheng B, Chambers TC, Kuo JF (1993). Cobra venom cardiotoxin (cytotoxin) isoforms and neurotoxin: comparative potency of protein kinase C inhibition and cancer cell cytotoxicity and modes of enzyme inhibition.. Biochemistry.

[11] Raynor RL, Zheng B, Kuo JF (1991). Membrane interactions of amphiphilic polypeptides mastoparan, melittin, polymyxin B, and cardiotoxin. Differential inhibition of protein kinase C, Ca2+/calmodulin-dependent protein kinase II and synaptosomal membrane Na,K-ATPase, and Na+ pump and differentiation of HL60 cells.. J Biol Chem.

[12] Wu PL, Lee SC, Chuang CC, Mori S, Akakura N (2006). Non-cytotoxic cobra cardiotoxin A5 binds to avb3 integrin and inhibits bone resorption. Identification of cardiotoxins as non-RGD integrin-binding proteins of the Ly-6 family.. J Biol Chem.

[13] Lee SC, Guan HH, Wang CH, Huang WN, Tjong SC (2005). Structural basis of citrate dependent and heparan sulfate-mediated cell surface retention of cobra cardiotoxin A3.. J Biol Chem.

[14] Sue SC, Brisson JR, Tjong SC, Huang WN, Lee SC (2001). Structures of heparin-derived disaccharide bound to cobra cardiotoxins: context-dependent conformational change of heparin upon binding to the rigid core of the three-fingered toxin.. Biochemistry.

[15] Tjong SC, Chen TS, Huang WN, Wu WG (2007). Structures of Heparin-Derived Tetrasaccharide Bound to Cobra Cardiotoxins: Heparin Binding at a Single Protein Site With Diverse Side Chain Interactions.. Biochemistry.

[16] Jayaraman G, Krishnaswamy T, Kumar S, Yu C (1999). Binding of nucleotide triphosphates to cardiotoxin analogue II from the Taiwan cobra venom (Naja naja atra). Elucidation of the structural interactions in the dATP-cardiotoxin analogue II complex.. J Biol Chem.

[17] Wang CH, Liu JH, Lee SC, Hsiao CD, Wu WG (2006). Glycosphingolipid-facilitated membrane insertion and internalization of cobra cardiotoxin. The sulfatide cardiotoxin complex structure in a membrane-like environment suggests a lipid-dependent cell-penetrating mechanism for membrane binding polypeptides.. J Biol Chem.

[18] Ishizuka I (1997). Chemistry and functional distribution of sulfoglycolipids.. Prog Lipid Res.

[19] Dufourcq J, Faucon JF, Bernard E, Pezolet M, Tessier M (1982). Structure-function relationships for cardiotoxins interacting with phospholipids.. Toxicon.

[20] Chen KC, Kao PH, Lin SR, Chang LS (2007). The mechanism of cytotoxicity by Naja naja atra cardiotoxin 3 is physically distant from its membrane-damaging effect.. Toxicon.

[21] Buckland AG, Wilton DC (2000). Anionic phospholipids, interfacial binding and the regulation of cell functions.. Biochim Biophys Acta.

[22] Stace CL, Ktistakis NT (2006). Phosphatidic acid- and phosphatidylserine-binding proteins. 23.. Biochim Biophys Acta.

[23] Schlegel RA, Williamson P (2001). Phosphatidylserine, a death knell.. Cell Death Differ.

[24] Fadok VA, Bratton DL, Frasch SC, Warner ML, Henson PM (1998). The role of phosphatidylserine in recognition of apoptotic cells by phagocytes.. Cell Death Differ.

[25] Fadeel B, Xue D (2009). The ins and outs of phospholipid asymmetry in the plasma membrane: roles in health and disease.. Crit Rev Biochem Mol Biol.

[26] Boas FE, Forman L, Beutler E (1998). Phosphatidylserine exposure and red cell viability in red cell aging and in hemolytic anemia.. Proc Natl Acad Sci USA.

[27] Zwaal RF, Comfurius P, Bevers EM (2005). Surface exposure of phosphatidylserine in pathological cells.. Cell Mol Life Sci.

[28] Kukhtina VV, Weise K, Osipov AV (2000). [MALDI-mass spectrometry for identification of new proteins in snake venoms].. Bioorg Khim (Russian).

[29] Jones G, Willett P, Glen RC, Leach AR, Taylor RD (1997). Development and Validation of a Genetic Algorithm for Flexible Docking.. J Mol Biol.

[30] Jones G, Willett P, Glen RC (1995). Molecular Recognition of Receptor Sites Using a Genetic Algorithm with a Description of Desolvation.. J Mol Biol.

[31] Efremov RG, Volynsky PE, Nolde DE, Arseniev AS (2001). Implicit two-phase solvation model as a tool to assess conformation and energetics of proteins in membrane-mimic media.. Theor Chem Acc.

[32] Pyrkov TV, Chugunov AO, Krylov NA, Nolde DE, Efremov RG (2009). PLATINUM: a web tool for analysis of hydrophobic/hydrophilic organization of biomolecular complexes.. Bioinformatics.

[33] Lindahl E, Hess B, van der Spoel D (2001). GROMACS 3.0: A package for molecular simulation and trajectory analysis.. J Mol Model.

[34] Efremov RG, Gulyaev DI, Vergoten G, Modyanov NN (1992). Application of three-dimensional molecular hydrophobicity potential to the analysis of spatial organization of membrane domains in proteins: I. Hydrophobic properties of transmembrane segments of Na+, K(+)-ATPase.. J Protein Chem.

[35] van Gunsteren WF, Billeter SR, Eising AA, Hünenberger PH, Krüger P (1996). Biomolecular Simulation: The GROMOS96 Manual and User Guide; vdf Hochschulverlag AG an der ETH Zürich and BIOMOS b.v.: Zürich, Groningen.

[36] Berendsen HJC, Postma JPM, van Gunsteren WF, Hermans J, Pullman B (1981). Interaction models of water in relation to protein hydration. Intermolecular Forces.

[37] Darden T, York D, Pederson L (1993). Particle Mesh Ewald: an N Log(N) method for Ewald sums in large systems.. J Chem Phys.

[38] DeLano WL (2002). The PyMOL Molecular Graphics System.. DeLano Scientific.

[39] Efremov RG, Nolde DE, Vergoten G, Arseniev AS (1999). A solvent model for simulations of peptides in bilayers. I. Membrane-promoting alpha-helix formation.. Biophys J.

[40] Efremov RG, Volynsky PE, Nolde DE, Dubovskii PV, Arseniev AS (2002). Interaction of cardiotoxins with membranes: a molecular modeling study.. Biophys J.

[41] Jorgensen WL (1989). Interactions between amides in solution and the thermodynamics of weak binding.. J Am Chem Soc.

[42] Sue SC, Jarrell HC, Brisson JR, Wu WG (2001). Dynamic characterization of the water binding loop in the P-type cardiotoxin: implication for the role of the bound water molecule.. Biochemistry.

[43] Chien KY, Chiang CM, Hseu YC, Vyas A, Gordon S (1994). Two distinct types of cardiotoxin as revealed by the structure and activity relationship of their interaction with zwitterionic phospholipid dispersions.. J Biol Chem.

[44] Sue SC, Chien KY, Huang WN, Abraham JK, Chen KM (2002). Heparin binding stabilizes the membrane-bound form of cobra cardiotoxin.. J Biol Chem.

[45] Gatineau E, Toma F, Montenay-Garestier T, Takechi M, Fromageot P (1987). Role of tyrosine and tryptophan residues in the structure–activity relationships of a cardiotoxin from Naja nigricollis venom.. Biochemistry.

[46] Gatineau E, Takechi M, Bouet F, Mansuelle P, Rochat H (1990). Delineation of the functional site of a snake venom cardiotoxin: preparation, structure, and function of monoacetylated derivatives.. Biochemistry.

[47] Stevens-Truss R, Hinman CL (1996). Chemical modification of methionines in a cobra venom cytotoxin differentiates between lytic and binding domains.. Toxicol Appl Pharmacol.

[48] Allen TM, Gregoriadis G (1984). Calcein as a tool in liposome methodology.. Liposome Technology.

[49] Sevcsik E, Pabst G, Richter W, Danner S, Amenitsch H (2008). Interaction of LL-37 with model membrane systems of different complexity: influence of the lipid matrix.. Biophys J.

[50] Gilquin B, Roumestand C, Zinn-Justin S, Menez A, Toma F (1993). Refined three-dimensional solution structure of a snake cardiotoxin: analysis of the side-chain organization suggests the existence of a possible phospholipid binding site.. Biopolymers.

[51] Hodges SJ, Agbaji AS, Harvey AL, Hider RC (1987). Cobra cardiotoxins. Purification, effects on skeletal muscle and structure/activity relationships.. Eur J Biochem.

[52] Wang PC, Loh KS, Lin ST, Chien TL, Chiang JR (2009). Consensus sequence L/PKSSLL mimics crucial epitope on Loop III of Taiwan cobra cardiotoxin.. Biochemical and Biophysical Research Communications.

[53] Dementieva DV, Bocharov EV, Arseniev AS (1999). Two forms of cytotoxin II (cardiotoxin) from Naja naja oxiana in aqueous solution: spatial structures with tightly bound water molecules.. Eur J Biochem.

[54] Sun YJ, Wu WG, Chiang CM, Hsin AY, Hsiao CD (1997). Crystal structure of cardiotoxin V from Taiwan cobra venom: pH-dependent conformational change and a novel membrane-binding motif identified in the three-finger loops of P-type cardiotoxin.. Biochemistry.

[55] Konshina AG, Volynskii PE, Arsen'ev AS, Efremov RG (2003). Interaction of cardiotoxin A5 with a membrane: role of conformational heterogeneity and hydrophilic properties.. Bioorg Khim.

[56] Polyansky AA, Ramaswamy R, Volynsky PE, Sbalzarini IF, Marrink SJ (2010). Antimicrobial Peptides Induce Growth of Phosphatidylglycerol Domains in a Model Bacterial Membrane.. J Phys Chem Lett.

[57] Polyansky AA, Volynsky PE, Arseniev AS, Efremov RG (2009). Adaptation of a membrane-active peptide to heterogeneous environment. II. The role of mosaic nature of the membrane surface.. J Phys Chem B.

[58] van den Eijnde SM, van den Hoff MJ, Reutelingsperger CP, van Heerde WL, Henfling ME (2001). Transient expression of phosphatidylserine at cell-cell contact areas is required for myotube formation.. J Cell Sci.

[59] Zachowski A (1993). Phospholipids in animal eukaryotic membranes: transverse asymmetry and movement.. Biochem J.

[60] Verkleij AJ, Zwaal RF, Roelofsen B, Comfurius P, Kastelijn D (1973). The asymmetric distribution of phospholipids in the human red cell membrane. A combined study using phospholipases and freeze-etch electron microscopy.. Biochim Biophys Acta.

[61] Sessions A, Horwitz AF (1983). Differentiation-related differences in the plasma membrane phospholipid asymmetry of myogenic and fibrogenic cells.. Biochim Biophys Acta.

[62] Bütikofer P, Lin ZW, Chiu DT, Lubin B, Kuypers FA (1990). Transbilayer distribution and mobility of phosphatidylinositol in human red blood cells.. J Biol Chem.

[63] Takechi M, Tanaka Y, Hayashi K (1986). Binding of cardiotoxin analogue III from Formosan cobra venom to FL cells.. FEBS Lett.

[64] Tzeng WF, Chen YH (1988). Suppression of snake-venom cardiotoxin-induced cardiomyocyte degeneration by blockage of Ca2+ influx or inhibition of non-lysosomal proteinases.. Biochem J.

[65] Bevers EM, Williamson PL (2010). Phospholipid scramblase: an update.. FEBS Lett.

[66] Scheuplein F, Schwarz N, Adriouch S, Krebs C, Bannas P (2009). NAD+ and ATP released from injured cells induce P2X7-dependent shedding of CD62L and externalization of phosphatidylserine by murine T cells.. J Immunol.

[67] Sharonov GV, Feofanov AV, Utkin YN, Arseniev AS Kirpichnikov MP (2008). Online application of scanning laser microscopy to study mechanisms of cytolysines action.. Innovative microscopy methods in science and education.

[68] Shao C, Novakovic VA, Head JF, Seaton BA, Gilbert GE (2008). Crystal structure of lactadherin C2 domain at 1.7A resolution with mutational and computational analyses of its membrane-binding motif.. J Biol Chem.

[69] Harris F, Dennison SR, Phoenix DA (2009). Anionic antimicrobial peptides from eukaryotic organisms.. Curr Protein Pept Sci.

