# Supplementary material for: Snake Cytotoxins Bind to Membranes via Interactions with Phosphatidylserine Head Groups of Lipids
Source: PLoS One. 2011 Apr 29;6(4):e19064. doi: 10.1371/journal.pone.0019064 (PMC3084733; doi:10.1371/journal.pone.0019064)
Supplement: Data S1 — Spatial models of CT 4 used in molecular docking. To take into account flexibility of the receptor (CT 4), docking of hPS was performed into a set of toxin conformations obtained via molecular dynamics (MD) and Monte Carlo (MC) simulations starting with the X-ray structure of CT A3 (PDB entry 1HOJ). Some details of MD protocol as well as the starting structures for MC search are indicated. Structural rearrangements of the toxin molecule in water (MD simulation) and membrane/water (MC search) environments are described. (DOC) [file pone.0019064.s001.doc]

### Supporting Information

*Data S1. Spatial models of CT 4 used in molecular docking*

To take into account flexibility of the receptor (CT 4), docking of hPS was performed into a set of toxin conformations obtained *via* molecular dynamics (MD) and Monte Carlo (MC) simulations starting with the X-ray structure of CT A3 (PDB entry 1HOJ).

***MD-models****.* MD simulations were carried out using the GROMOS96 force field **[1]** and the GROMACS 3.3 software [**2**]. CT molecule was placed in a water box (SPC model) with added counterions (Cl^-^). 3D Periodic boundary conditions were imposed. After the initial energy minimization (using 3000 steepest descent iterations), the system was heated from 5 to 300 K during 100 ps in NPT ensemble followed by 20 ns of MD collection run at 300 K. MD-conformations for subsequent docking of hPS were extracted with 1 ns interval along the last 10 ns of 20-ns MD trajectory (in total, 11 toxin structures). Similar to other CTs [**3**], in the course of MD runs backbone atoms of CT 4 exhibited substantial flexibility of the loop regions (residues 4-12, 27-35, 40-48) and only small fluctuations of the β-structured core. As a rule, loop II of CTs gives the main contribution to the overall flexibility of the toxin structure. Thus, for the resulting MD-conformations the average root-mean-square deviation (RMSD) from the starting structure for the backbone atoms of the β-structured core and the loop II region are 1.0 and 3.6 Ǻ, respectively. Such a plasticity of the loops is likely to provide the maximal solvent exposure of their apolar extremities. This facilitates deep insertion of the loops into the hydrophobic milieu of the cell membrane.

***MC-models.*** Above, we considered conformational abilities of the toxin molecule in water. On the other hand, binding of CT on the membrane/water interface may lead to some structural rearrangements of the protein, which are not specific for the bulk water environment. With this aim in view, several CT conformations mimicking its membrane-bound state were also used in docking simulations with hPS. The later ones were derived from MC simulations with an implicit membrane model (see Methods) and corresponded to the found low-energy states of the toxin. In MC calculations, the starting structures were the X-ray model and three MD conformations of CT 4 obtained in water (see above). All the resulting lowest-energy states revealed interactions with the hydrophobic membrane environment *via* the hydrophobic tips of the loops I-III. It should be noted that the overall 3D structure of the toxin is well retained after adsorption on the membrane/water interface. The most noticeable changes occurred in the membrane-binding motif which creates the so-called “hydrophobic bottom” [**4,5**]. Location of charged side chains of Lys and Arg residues 4, 5, 12, 23, 35, 36, and 50 forming a polar “belt” on the protein surface, was also modified to increase the accessible hydrophobic surface area capable of embedding into the membrane core. For the loop regions, backbone RMSDs between the resulting lowest-energy MC-states and the corresponding starting structures (X-ray and MD-models) were ~0.5 and ~1.5 Ǻ, respectively.

1. van Gunsteren WF, Billeter SR, Eising AA, Hünenberger PH, Krüger P, et al. Biomolecular Simulation: The GROMOS96 Manual and User Guide; vdf Hochschulverlag AG an der ETH Zürich and BIOMOS b.v.: Zürich, Groningen, 1996.

2. Lindahl E, Hess B, van der Spoel D. (2001) GROMACS 3.0: A package for molecular simulation and trajectory analysis. J Mol Model 7: 306-317.

3. Sun YJ, Wu WG, Chiang CM, Hsin AY, Hsiao CD (1997) Crystal structure of cardiotoxin V from Taiwan cobra venom: pH-dependent conformational change and a novel membrane-binding motif identified in the three-finger loops of P-type cardiotoxin. Biochemistry 36: 2403-2413.

4. Dauplais M, Neumann JM, Pinkasfeld S, Menez A, Roumestand C (1995) An NMR study of the interaction of cardiotoxin gamma from *Naja nigricollis* with perdeuterated dodecylphosphocholine micelles. Eur J Biochem 230: 213-220.

5. Dubovskii PV, Dementieva DV, Bocharov EV, Utkin YN, Arseniev AS (2001) Membrane binding motif of the P-type cardiotoxin. J Mol Biol 305: 137-149.
